# T-RAC: Study protocol of a randomised clinical trial for assessing the acceptability and preliminary efficacy of adding an exergame-augmented dynamic imagery intervention to the behavioural activation treatment of depression

**DOI:** 10.1371/journal.pone.0288910

**Published:** 2023-07-31

**Authors:** Alexandru Tiba, Marius Drugaș, Ioana Sârbu, Trip Simona, Carmen Bora, Daiana Miclăuș, Laura Voss, Ioana Sanislav, Daniel Ciurescu

**Affiliations:** 1 Department of Psychology, University of Oradea, Oradea, Romania; 2 The Hull York Medical School, University of York, York, United Kingdom; 3 Faculty of Medicine, Transilvania University, Brașov, Romania; PLoS ONE, UNITED STATES

## Abstract

**Background:**

Improving the existent effective treatments of depression is a promising way to optimise the effects of psychological treatments. Here we examine the effects of adding a rehabilitation type of imagery based on exergames and dynamic simulations to a short behavioural activation treatment of depression. We investigate the acceptability and the efficacy of an exergame-augmented dynamic imagery intervention added to behavioural activation treatment and associated mechanisms of change.

**Methods and analyses:**

In a two-arm pilot randomised controlled trial, the acceptability and preliminary efficacy of an exergame-augmented dynamic imagery intervention added to behavioural activation treatment for depressed individuals will be assessed. Participants (age 18–65) meeting criteria for depression are recruited by media and local announcements. 110 participants will be randomly allocated to behavioural activation plus imagery group or to standard behavioural activation group. The primary outcome is depressive symptom severity (Beck Depression Inventory II) and secondary outcomes are anhedonia, apathy and behavioural activation and avoidance. The outcomes are assessed at baseline, mid treatment, posttreatment and 3-month follow-up. Moderation and mediation analyses will be explored. An intention-to-treat approach with additional per-protocol analysis will be used for data analysis.

## Introduction

Major depressive disorder (MDD) is a common mental health problem that results in severe impairment of the life of affected individuals [1]. Although there are several effective treatments of depression, their efficacy was challenged, only a moderate-small effect being evidenced after removing the existing research biases [2]. Estimates suggest that 59% of depressed patients do not even respond to psychological treatments (50% reduction in symptoms) and two thirds do not remit [2]. To this shortcoming it adds the high rates of relapse, recurrence, residual symptoms, and the fact that many affected individuals receive no treatment. It is estimated that after treatment, 30 to 50% of recovered patients are still troubled by residual symptoms and up to 75% of patients relapse [3]. Improving the treatment for depression has a major impact on global mental health and economy.

Finding efficient ways to treat depression has always been a preoccupation for both researchers and clinicians. Despite significant efforts, the efficacy of treatments for depression did not significantly improve in time [4]. Targeting new mechanisms and optimising the already effective interventions are promising solutions to advance the treatment of depression [5].

Mental rehearsal interventions are considered promising stand-alone or added-on treatments for depression [5–9]. Recently, the effect of imagery has been tested by adding imagery to behavioural activation treatment for depression. Behavioural activation treatment is one of the most effective and robust psychological treatments for depression [10, 11]. Although adding imagery interventions to behavioural activation brings great promise for improving the treatment of depression, there are several potential improvements for imagery interventions. Renner et al. [9] have recently pointed out the limitations of mental imagery interventions in depression: only individuals who vividly imagine actions benefit from intervention; depressed individuals have difficulties vividly simulating actions, not knowing the ingredients of a prospective mental rehearsal of actions.

We suggest that a major source of potential improvements for mental imagery interventions comes from paying more attention to the contribution of the ongoing states of the sensorimotor and affective brain systems (motor, affective, and visual) to mental simulations. Considering the characteristics of the sensorimotor and affective brain systems involved in the mental construction of those experiences results in several advantages for imagery interventions. First, the differences in the states of the sensorial, affective, and motor systems recruited for simulation should be considered during imagery interventions for depressed individuals. For instance, depressed individuals show large differences in the state of modality-specific brain systems, having attenuated excitation of the motor cortex [12, 13] and deficient recruitment of brain systems involved in positive affect [14], which may influence the effect of the interventions. Second, considering how particular imagery modalities (e.g., visual versus motor) are used during the imagery process in depressed individuals may show a significant advantage. Differences in using some modalities over others may result in differences in the effect of imagery over behaviour [15]. For instance, the recruitment of the motor brain in imagery and the effect of mental imagery on action largely depend on the success of using kinesthetic rather than visual imagery [16]. Depressed individuals have more impaired motor imagery than visual imagery and compensate for this deficit by using modalities, which may limit the impact of imagery interventions [17]. Third, adopting an understanding of the mental simulation of feelings as mental re-activation of body sensations in imagery intervention may also bring a significant advantage for intervention. Recent research showed that felt sensations in the body are core components of feelings [18, 19], they can be maintained as representations in working memory [20], they are attenuated in depressed individuals [21], and that prospective simulations of sensations felt in the body during feelings in positive situations are attenuated in individuals with high levels of depressive symptomatology [22].

Here we propose that we may use these advantages and significantly improve imagery interventions by focusing on enhancing the embodiment of mental rehearsal in imagery-based treatments for depressed individuals. A large body of research suggests that the effect of mental simulations on emotion and behaviour heavily depends on their embodiments, or the efficient recruitment of specific sensorimotor and affective modalities in mental simulations by attention, verbal, and memory controls [23]. For instance, when simulating and paying attention to visual details rather than proprioceptive sensations, one will recruit the visual cortex for action simulation rather than the interoceptive and motor cortex, and vice versa [24]. Mental rehearsal of action is efficient for action and affective control when it adequately recruits motor and affective simulations [25]. Depressed individuals have difficulties recruiting motor and affective brain areas in their thinking. Thus, to optimise imagery treatment for depressed individuals, one should enhance the recruitment of modal (motor and affective) brain systems in the mental simulation process. When deficiencies of the modal brain areas required for imagery simulations are stable alterations (i.e., cognitive deficits of cognition), the intervention should follow a rehabilitation approach similar to motor rehabilitation imagery used in patients with brain damage (e.g., damages due to stroke). We focus on two potential solutions for improving imagery based on embodied processes: exergames and dynamic imagery.

A promising way to support the embodiment of simulations of action in depressed individuals is by using remote kinematics in the form of exergaming (XboxKinect^™^ games). Previous research showed that using exergaming results in the augmentation of motor (kinesthetic) imagery [26–28], increased motor rehabilitation in stroke patients [29], motor learning [30] and it is efficient as a stand-alone intervention for the reduction of depressive symptomatology [31–34]. Furthermore, exergaming has an increased acceptability and feasibility in various populations [29]. Based on these results, using exergaming as a tool for motor rehabilitation of action cognition in imagery interventions for depressed individuals seems to be worth investigating.

Another promising solution came from dynamic imagery. Dynamic imagery, as opposed to static imagery (mental rehearsal of movement without overt movement) requires mimicry of simulated movements [35]. Previous research has shown a *dynamic superiority effect*, individuals remembering better moving rather than static stimuli [36]. Similarly, a dynamic superiority effect has been evidenced for imagery. In comparison with static imagery, dynamic imagery results in higher vividness of mental imagery [37] motor learning [35] and behavioural performance [38]. Moreover, the imagery deficits which are evidenced in static motor imagery tasks are not evident when a dynamic strategy of motor imagery is applied for the same population [39].

Given that imagery methods added to behavioural activation treatments are promising for the treatment of depression [6, 9] and that dynamic imagery and stimulation by remote kinematic may help overcome limitations of existing imagery interventions, we intend to test the effect of adding a dynamic imagery intervention augmented by exergames to a short behavioural activation treatment [40].

Starting from the idea that action cognition and motor imagery deficits are stable alterations in depressed individuals [41–43] we use a rehabilitation type of motor imagery intervention based on principles of rehabilitation of motor imagery for individuals with neurological conditions. To this end, we integrate forward modelling of action and motor imagery [44], remote kinematics (Kinect) and embodied cognition account (action support of mental simulations, [25, 45]. This intervention is a new one that proved efficient in clinical work (Tiba, [Unpublished protocol]), [46]. There are several steps in the intervention. In the first session, the therapists introduce the *dynamic simulation routine*: how to support mental imagery of activities through gestures, language, and attention. Then, the patients undergo an exergame for 5 to 10 minutes and an “actfulness” exercise focusing on feelings of movement and motor simulation. After the exercise, the participants dynamically imagine a planned activity. In session two, patients are thought to restructure action memories by focusing on movements. Deficient action simulations are rehabilitated by (a) partial movements (they alternate covert with dynamic-partial movements simulations in response to stimuli); (b) linguistic supports (training in gerundival perceptions that is to recognize and name stimuli by actions; for instance, a door is recognized as a door to open; [47]), (c) enhanced perceptual and affective simulations (PETTLEP system, [48]), and (d) episodic memory support (they have to form future memories of *action cores*-last sequence of movement before perception of desired environmental change, correct them by experience, and remember at the end of the day). Thus, it is a rehearsal training that mixes actual and mental practice [49, 50] with augmentation of the motor component of simulations in thinking by enhancing *gestures*, *language*, *and episodic memory* as controls of simulation, and is applied to promote the use of motor simulations in everyday life.

We expect that adding a dynamic imagery intervention will augment the effects of behavioural activation treatment (BA) on severity of depressive symptoms. Second, we investigate whether adding the dynamic imagery intervention to behavioural activation treatment has any effect on secondary outcomes such as anhedonia, apathy, and vividness of imagery. Third, we investigate the moderators and mediators of the effects of the intervention.

## Methods and analysis

### Study design

**Fig 1:**
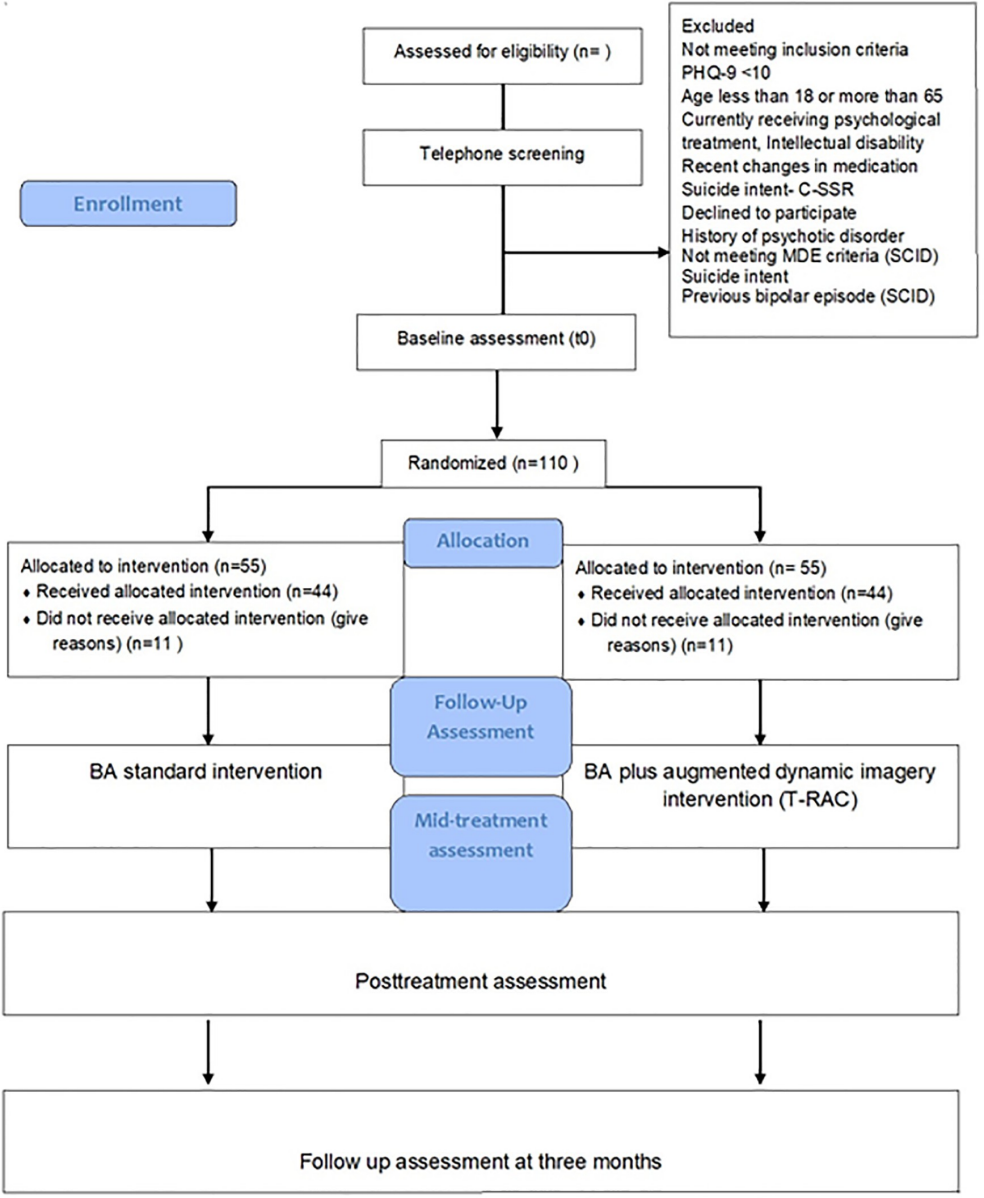
The study flow.

The study is a two-arm phase II randomised controlled trial (RCT) with a parallel design to compare a short behavioural activation (BA) treatment with BA plus dynamic imagery enhanced by exergames (XboxKinect^™^). We recruit participants by online media, advertisements, and by contacting local psychiatrists. After the online screening and baseline assessment, participants are randomised to either BA or BA plus T-RAC. Then the participants are enrolled in the treatment. Participants complete a weekly assessment, one assessment of mediators at week six, one assessment after treatment and one assessment at three months. The consort flow for the Study is presented in Fig 1.

#### Ethics and dissemination

This trial is approved by the ethics committee of the Faculty of Socio-Humanistic Sciences of University of Oradea (no: 2394/18.11.2022). Informed consent is required from participants before enrollment in the study. The results will be submitted for publication in peer-reviewed journals and presented at conferences.

*Trial registration number*. This trial was registered in the Clinical Trials Register: NCT05625230.

### Recruitment of participants

The recruitment of the participants will follow the CONSORT chart that will guide the flow of the participants. 110 participants will be recruited so that the study will have sufficient power to detect the differences at a 20% attrition rate. Participants will be selected through announcements about the study both online and at the local clinics. Participants are invited to complete an online screening questionnaire, including the PHQ-9 scale and questions for checking the eligibility criteria. Participants with a PHQ-9 score of ten or higher are programmed for the SCID-5-CV interview administered by telephone or face-to-face. Participants who are eligible will be randomly assigned (using Redcap) to one of the two groups: (1) behavioural activation (BA) or (2) BA plus rehabilitation of action simulations (BA Plus T-RAC).

Participants allocated to the behavioural activation arm will undergo a standard BA treatment. A therapist will administer the treatment for 8 sessions based on a manual built on the behavioural treatment protocol described by Martell et al. [51] and used in the COBRA trial [52]. Each session is focused on reviewing the activity log, discussing difficulties in the implementation of previously planned activities, planning “antidepressant” activities for the next week, and finding support for the implementation of the activity. Participants allocated to the BA plus T-RAC will undergo the same BA protocol plus an augmented imagery intervention. Each session is focused on reviewing the activity log, planning “antidepressant” activities for the next week, and finding support for the implementation of the activity. The difference is that after activity planning, the participant will follow Kinect for 10 minutes and will imagine one planned activity using actfulness. From session two, restructuring action memories are added.

### Eligibility

The inclusion criteria were age from 18 to 65, a score of 10 or higher on the PHQ-9 and a diagnosis of depression based on clinical interview. The exclusion criteria were: significant intellectual impairment; meeting criteria for a current psychotic or substance abuse disorder; history of mania/hypomania; significant changes in the dose of antidepressant medication during the past month; receiving psychological therapy; and the presence of suicidal intent.

### Management of suicidal ideation

We assess the suicidal ideation and intent at all assessments points of the study. Suicidal ideation is measured by the PHQ-9 item 9, BDI II, and clinical interview. If suicidal thoughts are reported, the C-SSRS interview is applied [53]. If suicidal intent (both unclear and clear) is identified the patient is not recruited in the study and contacted by phone by the clinical psychologist for proper recommendation to medical services. The participants with low risk suicidal ideation (suicidal ideation with no intent) are required to sign a non-suicide contract to continue the participation in the study. If suicide intent is identified, the participant is excluded from the study and necessary steps for medical assistance are followed (visit to psychiatrist, inpatient care). The suicide protocol is supervised by a licensed clinical psychologist.

### Outcomes

#### Primary outcome

*Depressive symptom severity* is assessed with the Beck Depression Inventory-II (BDI-II) [54]. BDI-II comprises 21 items assessing the DSM symptoms of the major depression episode during the past 2 weeks. Each question is scored on a 4-point Likert scale ranging from 0 to 3, with higher scores indicating higher depression. The BDI-II manual states that scores between 0 and 13 indicate minimal depression, 14 to 19 indicate mild depression, 20 to 28 indicate moderate depression, and 29 to 63 indicate severe depression [55]. Psychometric strengths such as validity and reliability were supported by numerous studies across many populations and cultural backgrounds [54, 56].

#### Secondary outcomes

*Depressive symptoms severity* is assessed with the Patient Health Questionnaire- 9 [57]. PHQ-9 scale contains nine items that measure the severity of nine depressive symptoms on a 4-point scale during the past two weeks. Each item is rated on a 4-point scale ranging from 0 (not at all) to 3 (nearly every day).

*Depression remission* is measured using The Structured Clinical Interview for DSM-5 Clinician Version [SCID-5-CV; 58], affective module at post measurement and follow-up. SCID-5 is a structured clinical interview used to determine the core diagnoses according to DSM-5 guidelines. Participants are considered remitted when they no longer fulfil diagnostic criteria for a major depression episode.

*Depression response* is assessed based on BDI II scores. To identify responders to treatment we compute the reliable change index. A reliable change index of more than 1.96 will classify a participant as a responder [59].

*Anhedonia severity* is assessed with the Snaith-Hamilton Pleasure Scale [60] SHAPS is a 14-item scale assessing the degree to which a person is able to experience pleasure or the anticipation of a pleasurable experience. Participants respond on a four points scale from strongly disagree to strongly agree. Each "disagree" response receives one point, whereas each "agree" response receives zero points. Thus, the final score ranges from 0 to 14.

*Apathy level* is assessed with the Apathy-Motivation Index [61]. AMI is a 18-item questionnaire used to identify three dimensions of apathy: Behavioural Activation (propensity to initiate goal-directed behaviour), Social Motivation (level of engagement in social interactions), and Emotional Sensitivity (feelings of positive and negative affection). Higher scores indicate greater apathy. Item scores are averaged to get scores for subscales and a total score (range 0–4) [61].

*Anxiety severity* is assessed with the Generalised Anxiety Scale 7 [GAD-7; 62]. GAD-7 is a self-report scale measuring the severity of generalised anxiety disorder (GAD) during the past 2 weeks. It contains seven items related to DSM criteria for anxiety. Participants respond on a 4-point scale. Each item is rated on a 4-point scale ranging from 0 (not at all) to 3 (nearly every day).

*Health and disability level outcome* is assessed with The World Health Organisation Disability Assessment Schedule (WHODAS 2.0) 12 –Self-Report Version WHODAS 12), [63]. WHODAS 12 is a practical, general assessment instrument. It has 6 subscale, each subscale containing two questions from the corresponding subscale in the full version: cognition, mobility, self-care, getting along, life activities and participation. The response options for each item ranged from 0 (none), to 4 (extreme/cannot do). The individual scores in each subscale are calculated by adding the two item scores. The total score ranges from 0–48.

*Adverse and unwanted effects of the experimental intervention* are assessed using the Negative Effects Questionnaire [64]. NEQ is a 20-item scale used to assess the adverse and unwanted effects of psychological treatments. Participants respond on a 5-point Likert-scale about the presence of adverse events during treatment such as symptoms (e.g., stress, shame, anxiety), quality (e.g., confidence regarding treatment and therapy), dependency (e.g., addiction to treatment medicine or therapy), stigma (e.g., feeling ashamed in front of other people), hopelessness, and failure (e.g., when thinking that things could get better) [65].

*The vividness of motor imagery* is assessed using the Vividness of Motor Imagery Questionnaire-2 (VMIQ-2) [66]. The vividness of imagining twelve movements is measured using a Likert scale from 1 (perfectly clear and vivid as you would normally picture the image) to 5 (no image or sensation at all, you are only aware you are “thinking” of the movement). The VMIQ-2 version was found to be as a psychometrically valid questionnaire) [66, 67].

*The level of rewards* is assessed with “The environmental and reward observation scale” (EROS); [68]. EROS consists of 10 items (e.g., “It is easy for me to find enjoyment in my life”), where responses are given on a Likert-type scale ranging from “completely disagree” to “absolutely agree”, on a 4-point scale. A higher score indicates a larger experience of reinforcing events from the environment.

*The level of rumination* is assessed with the Ruminative Response Scale-SV [69]. Participants respond on a 4-point Likert scale ranging from 1 (“almost never”) to 4 (“almost always”). The scale describes different components of the rumination response to depressed mood such as self-focused rumination (e.g., “I can’t stop thinking about some things”), symptom focused rumination (e.g., “I have never been able to distract myself from unwanted thoughts”) or rumination focused on the possible consequences and causes of the mood (e.g., "When I have a problem it will gnaw in my mind for a long time"). Brooding involves self-criticism, whereas reflection is related to more active problem solving.

*Activation and avoidance* is assessed using the Behavioral Activation for Depression Scale—Short Form (BADS-SF) [70]. BADS-SF is a 9-item scale used to assess the activation over the past week. It is organised in activation and avoidance subscales. BADS-SF is used to track when and how participants were activated during BA treatment.

*Working memory* is assessed with a Backward digit span task. Participants are given a series of digits and asked to repeat them backward. The score is based on how many numbers participants are able to repeat. A higher score indicates better working memory. Pairs of the sequences are read aloud to participants. The subsequent set of sequences will be one digit longer if both are successfully repeated and one digit shorter if none of the pair’s sequences is correctly repeated. The following pair of sequences will have the same length if one of the pair’s sequences is incorrectly repeated while the other sequence is correctly repeated [71].

*Executive functioning* is assessed with the verbal fluency test. In the verbal fluency test, participants are asked to say as many words as possible with a given letter (consonant F, A or S) for 1 minute.

*Verb fluency*. In the verb fluency task, participants are asked to produce as many verbs as possible for one minute. The participant’s score in each task is the number of total correct words.

*Acceptability ratings*. Acceptability is measured as satisfaction with the intervention, intention to continue and the intention to recommend the intervention assessed on a 5-points Likert scale. Drop-out rates are analysed as well.

*Affect and behaviour monitoring scale*. Changes in functioning, emotions, behaviour profile, efficacy, and difficulty of simulation are assessed. For each item, the score ranges from 1 (not at all) to 7 (very much).

### Proposed sample size

The sample size was established using G*Power and selecting the difference between two independent means. Forty-four participants per arm will be required to obtain a 90% statistical power. This estimation is based on previous studies targeting robust processes in depressed individuals (rumination) and between-treatment effect size of Cohen’s d = 0.7. Assuming a dropout rate of 20%, 55 patients are required in each treatment arm, resulting in a total of 110 participants.

### Statistical analyses

Multiple imputation methods will be selected to address the missing data. All randomised patients will be included in intention-to-treat analyses (ITT). Per-protocol analysis will be conducted separately. Analyses will be computed with an alpha significance level of 5% and two-tailed analyses. Analyses will be performed using IBM SPSS. Comparisons will be computed at a 5% significance level, and BDI II is the primary outcome variable considered as continuous variables. Differences between groups at the posttest will be analysed with a mixed ANOVA having group as the main effect and group *time as an interaction effect. Subgroup analyses will be conducted for anhedonia, apathy, motor imagery, and rumination. F-statistics and p values will be reported for main and interaction effects. The results will be considered as supporting evidence if the F test of the interaction effect is statistically significant (group*time interaction term). Potential mediators of treatment effects will be analysed exploratorily using Hayes model 4 analyses for the primary (depression severity) and secondary outcomes (anhedonia, and apathy). Hayes model 1 will be used to analyse the moderation. Between group effect sizes will be calculated based on Cohen’s d. Treatment completers will be analysed using per protocol analyses. Participants are considered "remitted" if they no longer fulfil the SCID-5 criteria for major depression episodes.

### Patient and public involvement

Participants and the public are not involved in the development of the study design. The intervention was modified based on piloting the procedures with healthy individuals and case studies of patients with depression. Based on the feedback from initial testing, procedures were modified to increase acceptability. Both XboxKinect^™^ exergames and imagery interventions were well received by various clinical and non-clinical populations in previous RCTs. Similarly, behavioural activation treatment is a well-received and efficient treatment of depression. Study results are disseminated through presentations at conferences and publications in major outlets for psychology. Local therapists will be trained to deliver behavioural activation. The public will know about the results available on the website and in the media.

## Discussion

The study is the first to evaluate an exergame-augmented dynamic imagery intervention added to behavioural activation treatment. It is expected that adding the exergame-augmented dynamic imagery intervention will increase the acceptability of the treatment and decrease the severity of depression, anhedonia, and apathy. We also expect, based on moderation analyses, that this effect to be more pronounced in participants with difficulties of motor imagery at baseline. We further investigate mediators of the treatment effect on the severity of depression and secondary outcomes.

This study is the first to investigate the added benefit of exergame-augmented dynamic motor imagery intervention to behavioural activation treatment. There is an expected advantage that this intervention will make the treatment more accepted (due to XboxKinect^™^) and will overcome the deficiencies in vividness of imagery and the recruitment of motor networks in imagery. If efficient it may work through different mechanisms than the standard BA treatment.

There are several strengths of the study: (1) it is a theory-driven intervention which could show an efficacy advantage, (2) it includes attractive exergames that may show additional benefits (exergame time may be increased and transformed into physical exercise) (3) we included a clinician administered semi-structured interview (SCID-5-CV), (4) we planned for a mid-treatment assessment to evaluate mechanisms of change. At session six, the participants will complete measures of the mechanisms of change, which will be carried out based on temporal precedence criteria [72], (5) assessors are blinded to participants’ treatment conditions.

There are several limitations of the study: (1) it is not a large scale RCT some limitations being related to the small sample size, (2) as the sample size was determined based on post-treatment differences, moderation and mediation analyses being underpowered, (3) participants with high risk of suicide will be excluded based on a score C-SSRS scale [53], limiting the generalisation of the results to participants with high risk of suicide.

This study investigates the acceptability, feasibility, and preliminary efficacy of adding an exergame-augmented dynamic imagery rehabilitative intervention to behavioural activation treatment. Thus, an ecological rehabilitative intervention for action cognition is available. This study increases our knowledge of highly available treatments with increased accessibility for individuals with depression.

## Supporting information

S1 Checklist SPIRIT 2013 checklist: Recommended items to address in a clinical trial protocol and related documents*. (DOC)

S1 Fig (DOC)

S1 File (PDF)

S2 File (PDF)
