# Supplementary material for: T-RAC: Study protocol of a randomised clinical trial for assessing the acceptability and preliminary efficacy of adding an exergame-augmented dynamic imagery intervention to the behavioural activation treatment of depression
Source: PLoS One. 2023 Jul 31;18(7):e0288910. doi: 10.1371/journal.pone.0288910 (PMC10389719; doi:10.1371/journal.pone.0288910)
Supplement: S1 Checklist — (DOC) [file pone.0288910.s001.doc]

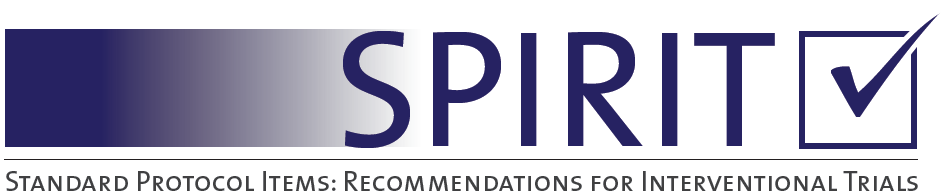


SPIRIT 2013 Checklist: Recommended items to address in a clinical trial protocol and related documents*

| Section/item | ItemNo | Description |
| --- | --- | --- |
| **Administrative information** | | |
| Title | 1 | T-RAC: Study Protocol of a Randomised Clinical Trial for Assessing the Acceptability and Preliminary Efficacy of Adding an Exergame-Augmented Dynamic Imagery Intervention to the Behavioural Activation Treatment of Depressed Individuals |
| Trial registration | 2a | Clinical Trials Register: NCT05625230 |
|  | 2b | - |
| Protocol version | 3 | 18.11.2022 NCT05625230 |
| Funding | 4 | This work was supported by a grant of the Ministry of Research, Innovation and Digitization, CNCS - UEFISCDI, project number PN-III-P1-1.1-TE-2021-1090, within PNCDI III and University of Oradea |
| Roles and responsibilities | 5a | Alexandru Tiba, University of Oradea-project administration, conceptualisation, funding acquisition, writing original draft, writing-review editing, resources; Laura Voss, University of York-conceptualisation, methodology, writing original draft; Ioana Sârbu, University of Oradea-investigation, project administration, writing-review editing; Marius Drugaș, University of Oradea- project administration, data analysis plans and monitoring; Carmen Bora, University of Oradea-methodology, supervision, validation, formal analysis, writing-review editing; Simona Trip, University of Oradea- writing-review editing; Daiana Miclăuș, University of Oradea-investigation, writing-review editing; Ioana Sanislav, University of Oradea- investigation, writing-review editing Daniel Ciurescu, University of Transilvania- writing-review editing. |
|  | 5b | Ministry of Research, Innovation and Digitization, CNCS – UEFISCDI, support@uefiscdi-direct.ro |
|  | 5c | The study sponsor and funders have no role in in study design; collection, management, analysis, and interpretation of data; writing of the report; and the decision to submit the report for publication nor they have ultimate authority over any of these activities |
|  | 5d | Not applicable |
| Introduction |  |  |
| Background and rationale | 6a | Finding efficient ways to treat depression has always been a preoccupation for both researchers and clinicians. Despite significant efforts, the efficacy of treatments for depression did not significantly improve in time (Cuijpers et al., 2020). Targeting new mechanisms and optimising the already effective interventions are promising solutions to advance the treatment of depression (Holmes et al., 2018).  Mental rehearsal interventions are considered promising stand-alone or added-on treatments for depression (Holmes et al., 2018; Pellas et al., 2022; Renner et al., 2017, 2019, 2021). Recently, the effect of imagery has been tested by adding imagery to behavioural activation treatment for depression. Behavioural activation treatment is one of the most effective and robust psychological treatments for depression (Cuijpers et al., 2007; Ekers et al., 2014). Although adding imagery interventions to behavioural activation brings great promise for improving the treatment of depression, there are several potential improvements for imagery interventions. Renner et al. (2021) have recently pointed out the limitations of mental imagery interventions in depression: only individuals who vividly imagine actions benefit from intervention; depressed individuals have difficulties vividly simulating actions, not knowing the ingredients of a prospective mental rehearsal of actions.  Here we propose that we may use these advantages and significantly improve imagery interventions by focusing on enhancing the embodiment of mental rehearsal in imagery-based treatments for depressed individuals.  A promising way to support the embodiment of simulations of action in depressed individuals is by using remote kinematics in the form of exergaming (XboxKinect™ games). Previous research showed that using exergaming results in the augmentation of motor (kinesthetic) imagery (Wriessnegger et al., 2014, 2018, 2020), increased motor rehabilitation in stroke patients (Almasi et al., 2022), motor learning (Sielużycki et al., 2019) and it is efficient as a stand-alone intervention for the reduction of depressive symptomatology (Abd-Alrazaq et al., 2022; Huang et al., 2022; Rica et al., 2020; Yunus et al., 2020). Furthermore, exergaming has an increased acceptability and feasibility in various populations (Almasi et al., 2022). Based on these results, using exergaming as a tool for motor rehabilitation of action cognition in imagery interventions for depressed individuals seems to be worth investigating.  Another promising solution came from dynamic imagery. Dynamic imagery, as opposed to static imagery (mental rehearsal of movement without overt movement) requires mimicry of simulated movements (Guillot et al., 2013). Previous research has shown a *dynamic superiority effect,* individuals remembering better moving rather than static stimuli (Mathews et al., 2007). Similarly, a dynamic superiority effect has been evidenced for imagery. In comparison with static imagery, dynamic imagery results in higher  vividness of mental imagery (Callow et al., 2006),  motor learning (Guillot et al., 2013) and behavioural performance (Guillot et al., 2021). Moreover,  the imagery deficits which are evidenced in static motor imagery tasks are not evident when a dynamic strategy of motor imagery is applied for the same population (Fusco et al., 2019). |
|  | 6b | Behavioral activation is a well-validated treatment of depression. Given that imagery methods added to behavioural activation treatments are promising for the treatment of depression (Pellas et al., 2022; Renner et al., 2021) and that dynamic imagery and stimulation by remote kinematic may help overcome limitations of existing imagery interventions, we intend to test the effect of adding a dynamic imagery intervention augmented by exergames to a short behavioural activation treatment (Jacobson et al., 2001). |
| Objectives | 7 | We expect that adding a dynamic imagery intervention will augment the effects of behavioural activation treatment (BA) on severity of depressive symptoms. Second, we investigate whether adding the dynamic imagery intervention to behavioural activation treatment has any effect on secondary outcomes such as anhedonia, apathy, and vividness of imagery. Third, we investigate the moderators and mediators of the effects of the intervention. |
| Trial design | 8 | The study is a two-arm phase II randomised controlled trial (RCT) with a superiority parallel design to compare a short behavioural activation (BA) treatment with BA plus dynamic imagery enhanced by exergames (XboxKinect™). We recruit participants by online media, advertisements, and by contacting local psychiatrists. After the online screening and baseline assessment, participants are randomised to either BA or BA plus T-RAC. Then the participants are enrolled in the treatment. Participants complete a weekly assessment, one assessment of mediators at week six, one assessment after treatment and one assessment at three months. |
| Methods: Participants, interventions, and outcomes | | |
| Study setting | 9 | The setting is at university Centre for Applied Research in Psychology, Romania. |
| Eligibility criteria | 10 | The inclusion criteria were age from 18 to 65, a score of 10 or higher on the PHQ-9 and a diagnosis of depression based on clinical interview. The exclusion criteria were significant intellectual impairment; meeting criteria for a current psychotic or substance abuse disorder; history of mania/hypomania; significant changes in the dose of antidepressant medication during the past month; receiving psychological therapy; and the presence of suicidal intent. |
| Interventions | 11 | Experimental: Behavior activation-PLUS T-RAC. Behavior activation/BA plus T-RAC Participants follow an 8 sessions intervention. Each session is focused on reviewing the activity log, generating antidepressant activities, planning the activities for the next week, and finding support for the implementation of the activity. After activity planning, the participants will follow an XboxKinect exergame for 10 minutes, an actfulness exercise and will imagine one planned activity using dynamic imagery. From session 2 restructuring action memories are added.  Active Comparator: Arm 2 Behavioral activation. Behavior activation/BA Participants in the behavioral activation arm will undergo a BA procedure. A therapist will administer an 8 sessions behavioral activation intervention based on the manual used in the COBRA trial. Each session is focused on reviewing the activity log, generating and planning anti-depressant activities for the next week, and finding support for the implementation of the activity. |
|  | 11b | Participant request, changes in current medication if this is the case, increase in suicide risk. |
|  | 11c | Session content will be verified for adherence to procols. Sessions will be chosen based on random selection. |
|  | 11d | Care as usual, medication. No psychotherapy is permitted during the trail. |
| Outcomes | 12 | ***Primary Outcome***  *Depressive symptom severity* is assessed with the Beck Depression Inventory-II (BDI-II), (Beck et al., 1996). BDI-II comprises 21 items assessing the DSM symptoms of the major depression episode during the past 2 weeks. Each question is scored on a 4-point Likert scale ranging from 0 to 3, with higher scores indicating higher depression.  ***Secondary outcomes***  *Depressive symptoms severity* is assessed with the Patient Health Questionnaire- 9 (Kroenke et al., 2001).  *Depression remission* is measured using The Structured Clinical Interview for DSM-5 Clinician Version (SCID-5-CV; First et al., 2016), affective module at post measurement and follow-up.  *Depression response* is assessed based on BDI II scores. To identify responders to treatment we compute the reliable change index. A reliable change index of more than 1.96 will classify a participant as a responder (Jacobson & Truax, 1991).  *Anhedonia severity* is assessed with the Snaith-Hamilton Pleasure Scale (Snaith et al., 1995)  *Apathy level* is assessed with the Apathy-Motivation Index (Ang et al., [201](https://bpspsychub.onlinelibrary.wiley.com/doi/full/10.1111/jnp.12262#jnp12262-bib-0001)7).  *Anxiety severity* is assessed with the Generalised Anxiety Scale 7 (GAD-7; Spitzer et al., 2006). GAD-7 is a self-report scale measuring the severity of generalised anxiety disorder (GAD) during the past 2 weeks.  *Health and disability level outcome* is assessed with The World Health Organisation Disability Assessment Schedule (WHODAS 2.0) 12 – Self-Report Version **(**WHODAS 12; Üstün et al., 2010). WHODAS 12 is a practical, general assessment instrument.  *Adverse and unwanted effects of the experimental intervention* are assessed using the Negative Effects Questionnaire (Rozental et al., 2019). NEQ is a 20-item scale used to assess the adverse and unwanted effects of psychological treatments.  *The vividness of motor imagery* is assessed using the Vividness of Motor Imagery Questionnaire-2 (VMIQ-2, [Roberts et al., 2008)](https://ipep.bangor.ac.uk/docs/Roberts et al. VMIQ-2 (2008).pdf).  *The level of rewards* is assessed with “The environmental and reward observation scale” (EROS; Armento & Hopko, 2007).  *The level of rumination* is assessed with the Ruminative Response Scale-SV (Treynor et al., 2003).  *Activation and avoidance* is assessed using the Behavioral Activation for Depression Scale - Short Form (BADS-SF; Manos et al., 2011).  *Working memory* is assessed with a Backward digit span task.  *Executive functioning* is assessed with the verbal fluency test. In the verbal fluency test, participants are asked to say as many words as possible with a given letter (consonant F, S or T) for 1 minute.  *Verb fluency.* In the verb fluency task, participants are asked to produce as many verbs as possible for one minute. The participant's score in each task is the number of total correct words.  *Acceptability ratings.* Acceptability is measured as satisfaction with the intervention, intention to continue and the intention to recommend the intervention assessed on a 5-points Likert scale. Drop-out rates are analysed as well.  *Affect and behaviour monitoring scale.* Weekly changes in functioning, emotions, behaviour profile, efficacy, and difficulty of simulation are weekly assessed. For each item, the score ranges from 1 (not at all) to 7 (very much) |
| Participant timeline | 13 | See diagram |
| Sample size | 14 | The sample size was established using G*Power and selecting the difference between two independent means. Forty-four participants per arm will be required to obtain a 90 % statistical power. This estimation is based on previous studies targeting robust processes in depressed individuals (rumination) and between-treatment effect size of Cohen’s d = 0.7. Assuming a dropout rate of 20 %, 55 patients are required into each treatment arm resulting in a total of 110 participants. |
| Recruitment | 15 | Psychiatry clinics are contacted for recruitment of patients. Media announcements are made. |
| **Methods: Assignment of interventions (for controlled trials)** | | |
| Allocation: |  |  |
| Sequence generation | 16a | RCap software was used generate the randomization of the participants. One member of the team generated the randomization sequence for all 110 participants. Only the research assistant allocated the participants to treatment groups. The research assistant had no direct contact to the person who randomized the sequence. After the participant was cleared for the eligibility criteria, the research assistant asked the PI for the group number to allocate the participant. The PI requested the allocation from the randomization staff. |
| Allocation concealment mechanism | 16b | No direct contact between the randomization staff and person who assigned participants. The sequence was communicated by telephone through the PI for each participant. |
| Implementation | 16c | DM generated the allocation sequence, BF enrols participants, and SI will assign participants to interventions |
| Blinding (masking) | 17a | Both outcome assessors and the participants are blinded. Assessors do not know the group of the therapist and participants. The research assistant asks for completing the assessment of the patient. |
|  | 17b | Participant may know the treatment condition at the end of the intervention. After intervention the participants may enrol in workshop that includes the add on imagery intervention. |
| **Methods: Data collection, management, and analysis** | | |
| Data collection methods | 18a | Each response is registered in an excel file by the assessors at the moment of data collection. This file is centralized by the research assistant. |
|  | 18b | Plans to promote participant retention and complete follow-up, including list of any outcome data to be collected for participants who discontinue or deviate from intervention protocols |
| Data management | 19 | Data are entered in a googleform for each assessment point based on responses of the participants and collected in an excel file. After entering the responses, the assessors do not have access to the data. The research assistant and the principal investigator have access to data for final centralisation. |
| Statistical methods | 20a | Preliminary group analyses, ANOVA , t tests, moderation and mediation based on Hayes models will be used. The analyses are mentioned in the protocol. |
|  | 20b | Moderation analyses using the Hayes model will be used for subgroup analyses. |
|  | 20c | Multiple imputation method will used for handling missing data. |
| **Methods: Monitoring** | | |
| Data monitoring | 21a | Data monitoring committee (DMC) was no required by the contract. The decision not to include a DMC was because the intervention is short, the intervention is based on psychological procedures that are well-validated and known for not harming participants, participants with critical indications are not included in the study. Similarly, the monitoring based on supervision should be enough for the safety of participants. Two experienced researchers monitor the procedures regarding the data collection and analysis. Both researchers are not involved in the data collection and intervention delivery. |
|  | 21b | The stopping guideline are dependent on the requiting the planned number of participants. Interim results are analysed to provide safety for participants which are monitored regarding their state, possible deterioration, and suicide thoughts. Two interim reports are required by the funding agency. |
| Harms | 22 | The staff that administer the intervention participate in a weekly supervision session. Any problems that may represent a harm are reported to an experienced clinical psychologist. The state of the participants is registered session-by-session and reasons for possible lack of reduction of symptoms are discussed. If any negative effects cannot be addressed a meeting with the clinical psychologist is scheduled to further analyse the problems and find appropriate reasons. At the end of the intervention the participants complete *The Negative Effects Scale* which measure the negative effects of psychological treatments. These will be reported in the publications. |
| Auditing | 23 | An independent (from both the funder and the investigators) financial auditing process is required each year. Frequency and procedures for auditing trial conduct, if any, and whether the process will be independent from investigators and the sponsor |
| Ethics and dissemination | | |
| Research ethics approval | 24 | The research received approval from the research ethics committee of the Faculty of Socio-Humanistic Sciences of University of Oradea (no: 2394/18.11.2022 |
| Protocol amendments | 25 | Changes in the research plans and protocol are communicated to the ethic committee and funding agency. |
| Consent or assent | 26a | The assessors obtain a signed informed consent after a face-to-face meeting before the first assessment. Participants also agree to complete the screening for selectin for the clinical interview. Who will obtain informed consent or assent from potential trial participants or authorised surrogates, and how (see Item 32) |
|  | 26b | No additional collection and use of participant data and biological specimens in ancillary studies is necessary |
| Confidentiality | 27 | Each participant received an identifier at the enrolment in the study. The access the enrolment list is possible only for the PI and the research assistant. Further assessments include only the personal identifier based on project acronym and a number Trac00X. |
| Declaration of interests | 28 | No financial and competing interests are declared for the principal investigators. |
| Access to data | 29 | The final data set will be accessed by the experienced researchers in the study. |
| Ancillary and post-trial care | 30 | Participants are monitored based on session-by-session measurement of their depressive state. Any changes in state that represent a risk for the participant (suicide risk) is signalled to be assessed by an experienced clinical psychologist. Participants during the selection phase with high risk of suicide are directed toward appropriate services. Based on previous studies we expect no harm due to the intervention. However, participants who report harm from following the program will be assessed by an experienced clinician to analyse the best care for the person. |
| Dissemination policy | 31a | The results are communicated through publications and conferences. Dissemination of the intervention to the mental health professionals is planned in the last part of the project. No publication restrictions exist. |
|  | 31b | No professional writers will be used. The authorship eligibility is based on sole contribution to the study, data analysis and management and writing the manuscripts. |
|  | 31c | Access is provided for the funder under the contractual specifications. Public access will be granted based on requests or based on specific publications. of the |
| Appendices |  |  |
| Informed consent materials | 32 | Model consent form and other related documentation given to participants and authorised surrogates |
| Biological specimens | 33 | Not applicable/Plans for collection, laboratory evaluation, and storage of biological specimens for genetic or molecular analysis in the current trial and for future use in ancillary studies, if applicable |

*It is strongly recommended that this checklist be read in conjunction with the SPIRIT 2013 Explanation & Elaboration for important clarification on the items. Amendments to the protocol should be tracked and dated. The SPIRIT checklist is copyrighted by the SPIRIT Group under the Creative Commons “[Attribution-NonCommercial-NoDerivs 3.0 Unported](http://www.creativecommons.org/licenses/by-nc-nd/3.0/)” license.
