# Supplementary material for: T-RAC: Study protocol of a randomised clinical trial for assessing the acceptability and preliminary efficacy of adding an exergame-augmented dynamic imagery intervention to the behavioural activation treatment of depression
Source: PLoS One. 2023 Jul 31;18(7):e0288910. doi: 10.1371/journal.pone.0288910 (PMC10389719; doi:10.1371/journal.pone.0288910)
Supplement: S1 Fig — (DOC) [file pone.0288910.s002.doc]

Figure 1

|  | **STUDY PERIOD** | | | | | | | |
| --- | --- | --- | --- | --- | --- | --- | --- | --- |
|  | **Enrolment** | **Allocation** | **Post-allocation** | | | | | **Close-out** |
| **TIMEPOINT**** | ***Week 0*** | **Week 0** | ***Week1*** | ***Week 6*** | ***AfterWeek 8*** |  |  | ***At three months*** |
| **ENROLMENT:** |  |  |  |  |  |  |  |  |
| **Eligibility screen** | X |  |  |  |  |  |  |  |
| **Informed consent** | X |  |  |  |  |  |  |  |
| **Allocation** |  | X |  |  |  |  |  |  |
| **INTERVENTIONS:** |  |  |  |  |  |  |  |  |
| ***[Behavioral Activation - Trac]*** |  |  |  |  |  |  |  |  |
| ***[Behavioral Activation]*** |  |  | X |  | X |  |  |  |
| **ASSESSMENTS:** |  |  |  |  |  |  |  |  |
| *BDI-II* |  | X |  |  | X |  |  | X |
| *PHQ-9* |  | X |  | X | X |  |  | X |
| *SCID-5-CV* |  | X |  | X | X |  |  | X |
| *Anhedonia & apathy* |  | X |  |  | X |  |  | X |
| *Anxiety severity* |  | X |  |  | X |  |  |  |
| *Health and disability level* |  | X |  |  | X |  |  |  |
| *The vividness of motor imagery* |  | X |  | X | X |  |  |  |
| *Working memory, Fluency & Affect and behavior monitoring* |  | X |  | X | X |  |  | X |
| *Negative Effects & Acceptability* |  |  |  | X | X |  |  | X |

*Recommended content can be displayed using various schematic formats. See SPIRIT 2013 Explanation and Elaboration for examples from protocols.

**List specific timepoints in this row.
