# Supplementary material for: T-RAC: Study protocol of a randomised clinical trial for assessing the acceptability and preliminary efficacy of adding an exergame-augmented dynamic imagery intervention to the behavioural activation treatment of depression
Source: PLoS One. 2023 Jul 31;18(7):e0288910. doi: 10.1371/journal.pone.0288910 (PMC10389719; doi:10.1371/journal.pone.0288910)
Supplement: S1 File — (PDF) [file pone.0288910.s003.pdf]

PROJECT TITLE: STUDY TESTING THE EFFECTIVENESS OF THE ADDITION OF A MOTOR COGNITIVE REHABILITATION INTERVENTION IN DEPRESSION (Study 2)
PROJECT PN-III-P1-1.1-TE-2021-1090 Testing the effectiveness of a psychological treatment for the rehabilitation of motor cognition in depression (T-RAC)

Project director: Tiba Alexandru
Phone: 0754598233
Email: alexandrutiba@gmail.com
Research assistant: Sîrbu Ioana
Phone: 0748194311
Email: ioana.uoradea@gmail.com

I. Brief Description of the Purpose of the Project:

The study is carried out within the Department of Psychology, University of Oradea and is conducted under the coordination of the university lecturer. Dr. Alexandru Tiba. The aim of this study is to investigate the effect of adding a motor cognition rehabilitation intervention to behavioral activation treatment on the level of depression, acceptability, feasibility, anhedonia and motivation as well as mediating variables.

II. Specifying the planned dates for the start and completion of the project:
15.01.2023-31.05.2024

III. Number of participants:
In order to estimate the sample size, to detect an effect of medium size ($\alpha = 0.05$, effect size= 0.90), for 2x2 mixed ANOVA, a total sample of 110 participants, (n=55 each group) (G*Power).

IV. Specifying the characteristics of the resources used in the research activity
HR:
- 110 participants and project members
- research participation consent forms for the subjects involved in the study experimental

Material resources: psychometric instruments, SCID-5-CV, audio-video equipment.

V. Recruitment method:
Participants will be recruited from the general population and screened based on identification of depression level (low or high), then assigned to high depression and low depression groups. Prescreening will be based on the PHQ-2 (two questions related to loss of interest and depressive states). If the answer to the two questions adds up to a score of 3 or higher, the participant will be allocated for screening. Will be called/scheduled for screening. The PHQ-9 will be administered by telephone and the SCID 5 interview for affective modules and exclusion criteria. If eligible, the participant will be placed on the participant list and randomly assigned to one of the two groups. The selection will be made in two waves in wave 1 from January 2023 to August 2023 and in wave 2 from September 2023 to May 2024.

Exclusion criteria: hospitalization in the last month for psychiatric treatment, manifestation of psychotic episodes, manifestation of suicidal behavior, consumption of psychoactive substances.

VI. Description of the research method and design of the project:

Theoretical premise: Starting from evidence of deficits in action cognition and motor imagery in depressed individuals (Bortolato et al., 2016; Chen et al., 2013; Gorwood et al., 2014) we developed a rehabilitative motor imagery training. This intervention is based on the principles of motor imagery rehabilitation (in sports and in the field of neurorehabilitation), on motor cognition rehabilitation procedures in individuals with neurological conditions (Shadmehr, Smith, Krakauer, 2010), remote kinematics studies (Kinect) and anchored cognition (action)—the support of mental simulations, Glenberg, 2010). It is a new add-on intervention that has been shown to be effective in clinical practice (Tiba & Manea, 2022; Tiba, unpublished protocol).
In this study, the intervention is added to an 8-session behavioral activation treatment. There are several stages of the intervention. The structure of the first intervention includes: (1) the therapist explains the intervention; (2) the therapist demonstrates the dynamic/augmented simulation skills (dynamic imagery) and repeats it with appropriate feedback to the patient, (3) the participant follows kinect training for 10 minutes, (4) follows the ACTFULNESS exercise and practices the exercise for 12 minutes in which they practice augmented—simulations in response to perceptions (in the contexts in which they perform activities). In the second session he learns dynamic memorization of daily activities and at the end of each day he will recall a memorized activity.
The intervention was built on two well-known interventions: mindfulness meditation-body scanning and dysfunctional thought recording, but adapted to balance action simulations in mental responses to perceptions, difficult situations, and negative situations. Deficient action simulations are rehabilitated by (a) partial movements (alternate covert simulations with dynamic-partial movements in response to stimuli); (b) linguistic supports (training in gerundive perceptions Lambie & Marcel, 2002 - for example, recognizing and naming stimuli through actions - a door to open), (c) enhanced perceptual and affective simulations (PETLEP system, Holmes & Collins, 2001) , and (d) supporting episodic memory (must form future memories of action nuclei - the last movement sequence before the perception of the desired environmental change, correct them through experience and recall them at the end of the day). Thus, it is a training that includes a mixed (mental and actual) mental repetition practice (Courtine, Papaxanthis, Gentili, & Pozzo, 2004; Malouin, Jackson, & Richards, 2013) with increasing the motor component of simulations in thinking by improving gestures, language and episodic. memory as simulation controls and is applied to promote the use of motor simulations in everyday life.

Procedure:
A pilot study testing the feasibility, acceptability, and effectiveness of adding imagery intervention to standard behavioral activation intervention. Recruitment of participants will follow the CONSORT chart that will guide the flow of participants. 110 participants will be recruited so that the study is sufficiently powered to detect differences when dropout rates of 20% are expected. Participants will be recruited through study announcements both online and at local clinics (in collaboration with several psychiatrists). They will be selected based on a telephone interview regarding the level of depression (PHQ-9 ≥10 meeting criteria for SCID-5 depressive episode). After inclusion in the study, the initial assessment T0 will be done, which consists of administering the tests for the measured variables.

After baseline assessment subjects will be randomized (using www.randomizer.org) to one of two groups: (1) standard behavioral activation (BA) and (2) BA Plus (T-RAC). At the sixth meeting, the intermediate evaluation of the mediators (ruminations, working memory, anhedonia, and apathy-loss of interest) will be carried out. The end-of-treatment assessment will be done one week after the end of (T1). The follow-up evaluation will be done after 3 months (T2).

Participants will be voluntary individuals from the general population willing to participate who meet the selection criteria for the study.

Design: 2X2 mixed-randomized two-arm clinical trial.

Tools used:
Acceptability- Visual analogue scale for level of satisfaction with the intervention and whether they would recommend the intervention to others.
Feasibility-indicators related to drop-out rate, no-show, adverse effects (Negative Events Scale-Negative Effects Scale (NEQ, Rozenthal and Carlbring, 2015)
Anhedonia Level-Temporal Experience of Pleasure Scale (TEPS), (Gard, Germans Gard, Kring, & John, 2006).
Depression Level-QIDS-SR (Rush et al., 2003) has 16 items that measure depression level.
Apathy-subscore QIDS item 13/Index of apathy (3 items, Roberts et al., 2002).
Hamilton Scale for Depression (HRSD; Hamilton, 1960; Williams, 1998).
SCID 5 Interview for Affective Modules (SCID-5-; First et al., 2015).

Several questionnaires will be used: PHQ-9 (Patient Health Questionnaire-9) to measure the level of depression, VMIQ-R to establish the ability to imagine (Williams et al. 2012). Specific self-report scales for assessing motor imagery associated with action and affective states. BADS- The behavioral activation for depression scale (BADS, Kanter et al., 2007); Spontaneous Use of Imagery Scale (SUIS) (Reisberg, Pearson, & Kosslyn, 2003);

Mediators: During the intervention, the change in scores will be measured for (1) pleasure associated with movement during the intervention (2) anhedonia (3) apathy, (4) working memory-inverse digit span test, (5) verbal/actional fluency (6) affective states (7) vividness and ease of motor imagery, (8) rumination, (9) behavioral activation, (10) level of reinforcement.

Statistical analysis: A 2X2 mixed ANOVA design will be used for the effect of the group (BA, BA-PLUS and the moment of the pre-postintervention evaluation (intersubjects)) - on the outcome variables: anhedonia, interest, accessibility, feasibility (significance level: α=0.05, two -tailed).Secondary analyzes to ensure the robustness of the results will include analysis of differences in means (pre-post) between groups.

Secondary objectives: Linear regression using PROCESS macro model 1 of A. F. Hayes (2013) will be used for moderation. Mean, standard deviation, effect size and other statistics will be reported.

Description of the activities that the participants must and do (consent, debriefing, etc.):

During the research, the participants complete and express their agreement regarding participation (Annex 5 of REC_UO/2019) and follow the selection procedure. After

randomization, participants in the standard BA treatment group undergo 8 sessions of behavioral activation. Participants in the BA-PLUS group follow the same sessions with the addition of the T-RAC intervention. The behavioral activation intervention follows the protocol validated in efficacy studies - Martell et al. (2001; 2010) and COBRA (Richards et al., 2017).

VIII. Specifying the estimated time for subjects to complete the study requirements:
60 minutes/week for 8 weeks. Assessment sessions 60 minutes. Time variations will depend on the therapeutic need for the participants.

IX. Presentation of possible risks from a moral perspective, possible situations of discomfort or stress and specifying the measures taken to minimize them:
There are no anticipated risks to participants during or following participation in this study.
Participants in the experimental group follow a treatment validated on a large scale in both the European and North and South American populations. The protocol follows the BA, Martell et al., (2001;2010) and COBRA guidelines, being verified by COBRA project clinicians (McKey Dean, Voss Laura). Participants in both groups follow the behavioral activation protocol. The T-RAC intervention is added to the BA plus group. This intervention involves 10 minutes of Kinect exposure (a Kinect game for 10 minutes) that has a high level of acceptability across a diverse population (non-clinical and clinical) and a 12-minute audio-recorded imaging exercise. Similar imagery exercises have previously been tested both as a stand-alone intervention (Blackwell et al., 2015) and as an adjunct to BA (Patel et al., 2022) with high acceptability. Similar dynamic imaging exercises have also been tested in the normal population and shown to be effective. From the second session, the participants memorize one daily activity through the motor augmentation method and remember the created memory in the evening. Reminder interventions have been successfully tested in depressed patients.
In case of negative effects, the project director Alexandru Tiba will be notified. He will contact the participant and decide the course of action-reorientation towards other services appropriate to the situation and/or psychiatric consultation. Negative changes in emotional state will be addressed according to the protocols in force. Participants with a high risk of suicide are not eligible for the project, being directed to the appropriate services (Iova Sorin psychiatric office/psychiatry outpatient clinic).

Project director:
Name: Tiba Alexandru Date:
Signature:________________________
Afiliere:

Department of Psychology
University of Oradea

INFORMED CONSENT

Project title: Study 1 T-RAC-psychological intervention for depression
Project director: Tiba Alexandru
Research assistant: Sîrbu Ioana
The purpose of the research: The study is carried out within the Department of Psychology, University of Oradea and is carried out under the coordination of the university lecturer. Dr. Alexandru Tiba.
The purpose of this study is to compare two psychological interventions for depression.
Research description:
After selection into the study, you will fill out some questionnaires and have an interview to assess your general condition and ability to imagine. You will be assigned to one of two psychological intervention groups. A psychologist will contact you and you will be scheduled for the first of 8 weekly sessions. In these meetings you will follow your condition and the changes that have occurred, you will discuss with the psychologist the antidepressant activities that you can do and you will find solutions to support and achieve them. Between sessions you will have to do the anti-depressant activities and apply the things that support you to be able to achieve them. Midway through the program you will complete several questionnaires to track the changes you have made. Likewise, one week after the end of the program you will follow the evaluation of the results as well as 3 months after the program. The sessions will be audio recorded and randomly checked to ensure the fidelity of the intervention. Participation in the program means your agreement to record the sessions.
We ensure the confidentiality of your participation and the information provided during the study, as well as the safety of the procedures used. Your data will only be used for research purposes.
The questions are not defamatory or contain content that can produce negative emotions. The assessment is not a personal assessment and no value judgments will be made based on these responses. At any time you can opt out of participating in the research. You can opt-in to be informed about the results of the assessment by ticking the box corresponding to the request to provide information with yes. Upon application you will be sent your situation and appropriate recommendations for further assessment. Once you agree and consent to participate, you will be able to participate in the research.
Potential risks: There are no risks from participating in this study.
Potential benefits: If you opt-in for information about your condition you will receive an email in which results will be posted regarding this.
Costs / Compensation: There are also no financial or material benefits from participating in the study. Participation in this study is not financially or materially compensated
Contact person: alexandrutiba@gmail.com If you have additional questions about the research, you can always contact the project manager at alexandrutiba@gmail.com.
Confidentiality: Information collected will be kept confidential in accordance with applicable law. The data will be analyzed anonymously and will only be accessed by the research team. Based on Regulation (EU) 2016/679 on the protection of natural persons with regard to the processing of personal data and on the free movement of such data and repealing Directive 95/46/EC (General Data Protection Regulation) and Law no. . 506/2004 on the processing of personal data and the protection of privacy, the data provided by you in this study will only be used for the specified purpose of studying the role of cognitions and emotions and their interactions in the risk for emotional disorders and dysfunctional emotions and analysis of these factors. Statistical analyzes will be performed at the sample level and not at the individual level.
Voluntary Participation: Participation in this research is voluntary. The decision not to participate or to withdraw along the way will not result in any penalties, fines or loss of your benefits. However, if you wish to withdraw from the study you will be asked about the

reasons for your withdrawal. Participation may be terminated if: (1) your condition worsens, (2) situations arise related to ineligibility for the study (3) you do not perform the tasks assumed for the conduct of the program or do not attend 3 meetings.

Ethics Commission approval

This research was analyzed and approved by the Ethics Committee of the University of Oradea.

Signatures

Date________________ Date_______________

Project Director Study Participant

Name__________________ Name__________________

Signature______________ Signature__________
