# Supplementary material for: T-RAC: Study protocol of a randomised clinical trial for assessing the acceptability and preliminary efficacy of adding an exergame-augmented dynamic imagery intervention to the behavioural activation treatment of depression
Source: PLoS One. 2023 Jul 31;18(7):e0288910. doi: 10.1371/journal.pone.0288910 (PMC10389719; doi:10.1371/journal.pone.0288910)
Supplement: S2 File — (PDF) [file pone.0288910.s004.pdf]

PREZENTARE SUCCINTĂ A CONȚINUTULUI PROIECTULUI

TITLUL PROIECTULUI: STUDIU TESTARE EFICIENTAADĂUGĂRII UNEI INTERVENȚII DE REABILITATE A COGNIȚIEI MOTORII ÎN DEPRESIE (Studiul 2)
PROIECT PN-III-P1-1.1-TE-2021-1090 Testarea eficienței unui tratament psihologic de reabilitare a cogniției motorii în depresie (T-RAC)

Director proiect: Tiba Alexandru
Tel mobil: 0754598233
Email: alexandrutiba@gmail.com
Asistent cercetare: Sîrbu Ioana
Tel mobil: 0748194311
Email: ioana.uoradea@gmail.com

I. Descrierea succintă a scopului proiectului:
Studiul se desfășoară în cadrul Departamentului de Psihologie, Universitatea din Oradea și este derulat sub coordonarea dlui lector univ. dr. Alexandru Tiba. Scopul acestui studiu vizează investigarea efectului adăugării unei intervenții de reabilitare a cogniției motorii la tratamentul de activare comportamentală asupra nivelului depresiei, acceptabilității, fezabilității, anhedoniei și motivației precum și a variablilelor mediatoare.

II. Precizarea datelor planificate pentru începerea și finalizarea proiectului:
15.01.2023-31.05.2024

III. Numărul participanților:
În vederea estimării dimensiunii eșantionului, pentru a detecta un efect de mărime medie (α = 0,05, mărime efect= 0,90), pentru 2x2 mixt ANOVA, ar fi necesar un eșantion total de 110 de participanți, (n=55 fiecare grup) (G*Power).

IV. Precizarea caracteristicilor resurselor utilizate in activitatea de cercetare
Resurse umane:
- 110 participanți și membrii proiectului
- formulare de consimțământ al participării la cercetare pentru subiecții implicați în studiul experimental

Resurse materiale: instrumente psihometrice, SCID-5-CV, echipament audio-video.

V**. Prezentarea metodei de recrutare a participanților**:
Participanții vor fi recrutați dintre din populația generală și selectați pe baza identificării nivelului de depresie (scăzut sau ridicat), după care alocați în grupul cu nivel ridicat de depresie și cu nivel scăzut de depresie. Prescreeningul se va realiza pe baza PHQ-2 (două întrebări legate de pierderea interesului și stările depresive). Dacă răspunsul la cele două întrebări însumează un scor de 3 sau mai mare participantul va fi alocat pentru screening. Va fi sunat/programat pentru screening. Se va administra PHQ-9 telefonic și interviul SCID 5 pentru module afective și criteriile de excludere. Dacă este eligibil participantul va fi trecut pe lista participantilor și va fi alocat în mod randomizat unuia dintre cele două grupuri. Selecția se va face în două valuri în

valul 1 din luna ianuarie 2023 pană în august 2023 și în valul 2 din septembrie 2023 până în mai 2024.

Criterii de excludere: spitalizarea în ultima lună în vederea unui tratament psihiatric, manifestarea unor episoade psihotice, manifestare comportament suicidar, consumul de substanțe psihoactive.

VI. Descrierea metodei și designului de cercetare ale proiectului:

Premisa teoretică: Porning de la evidențierea deficitelor în cogniția acțională și de imagerie motorie la indivizii depresivi (Bortolato și colab. 2016; Chen și colab., 2013; Gorwood și colab., 2014) am dezvoltat un antrenament de imagerie motorie de tip reabilitare. Anceastă intervenție este bazată pe principiile de reabilitare a imaginilor motorii (în sport și în domeniul neuroreabilitarii), pe procedurile de reabilitare a cognitiție motorii la indivizi cu afecțiuni neurologice (Shadmehr, Smith, Krakauer, 2010), studiile de cinematică la distanță (Kinect) și cogniție ancorată (acțiune) -suportul simulărilor mentale, Glenberg, 2010). Este o nouă intervenție de tip add-on care s-a dovedit eficientă în practica clinică (Tiba & Manea, 2022; Tiba, protocol nepublicat).

În acest studiu, intervenția este adăugată la un tratament de activare comportamentală de 8 ședințe. Există mai multe etape ale intervenției. Structura primei intervenții include: (1) terapeutul explică intervenția; (2) terapeutul demonstrează abilitățile de simulare dinamică/ augmentate (imagerie dinamică) și o repetă oferind pacientului un feedback adecvat, (3) participantul urmează un antrenament kinect timp de 10 minute, (4) urmează exercițiul ACTFULNESS și exersează exercițiul timp de 12 minute în care exersează augmentat—simulări ca răspuns la percepții (în contextele în care desfășoară activități). În ședința a doua învață memorarea dinamică a activităților zilnice și la sfârșitul fiecărei zi își va reaminti o activitate memorată.

Intervenția a fost construită pe baza a două intervenții binecunoscute: meditația mindfulness-scanarea corpului și înregistrarea gândurilor disfuncționale, dar adaptată pentru a echilibra simulările de acțiune în răspunsurile mentale la percepții, situații dificile și situații negative. Simulările de acțiuni deficitare sunt reabilitate prin (a) mișcări parțiale (alternează simulări ascunse cu mișcări dinamice-parțiale ca răspuns la stimuli); (b) suporturi lingvistice (antrenament în percepțiile gerundivale Lambie & Marcel, 2002 - de exemplu, recunoașterea și denumirea stimulilor prin acțiuni - o ușă de deschis), (c) simulări perceptive și afective îmbunătățite (sistemul PETTLEP, Holmes & Collins, 2001), și (d) susținerea memoriei episodice (trebuie să formeze amintiri viitoare ale nucleelor de acțiune - ultima secvență de mișcare înainte de percepția schimbării dorite de mediu, să le corecteze prin experiență și să le amintească la sfârșitul zilei). Astfel, este un antrenament care include o practică de repetare mentală mixtă (mentală și actuală) (Courtine, Papaxanthis, Gentili și Pozzo, 2004; Malouin, Jackson și Richards, 2013) cu creșterea componentei motorii a simulărilor în gândire prin îmbunătățirea gesturilor, limbajului și episodic. memoria ca controale ale simulării și este aplicată pentru a promova utilizarea simulărilor motorii în viața de zi cu zi.

Procedură:
Un studiu pilot de testare a fezabilității, acceptabilității și eficienței adăugării intervenției de imagerie la intervenția standard de activare comportamentală. Recrutarea participanților va urma diagrama CONSORT care va ghida fluxul participanților. Vor fi recrutați 110 participanți, astfel încât studiul să aibă suficientă putere pentru a detecta diferențele atunci când se așteaptă rate de abandon de 20%. Participanții vor fi selectați prin anunțuri despre studiu atât online, cât și la clinicile locale (în colaborare cu mai mulți psihiatri). Aceștia vor fi selectați pe baza unui

interviu telefonic cu privire la nivelul depresiei (PHQ-9 ≥10 indeplinirea criteriilor pentru episod depresiv SCID-5). După includerea în studiu se va face evaluarea inițială T0 care constă în administrarea testelor pentru variabilele măsurate.

După evaluarea inițială subiecții vor fi randomizați (folosind www.randomizer.org) în unul dintre cele două grupuri: (1) activare comportamentală standard (BA) și (2) BA Plus (T-RAC). La a șasea ședință se va realiza evaluarea intermediară a mediatorilor (ruminații, memoria de lucru, anhedonie, și apatie-pierderea intersului). Evaluarea de la finalul tratamentului se va face la o săptămână după terminarea lui (T1). Evaluarea follow-up se va face după 3 luni (T2).

Participanții vor fi persoane voluntare din populația generală care doresc să participe care îndeplinesc criteriile de selecție pentru studiu.

Design: 2X2 mixt-studiu clinic randomizat cu două brațe.

Instrumente utilizate:
Acceptabilitate- Scala analoagă vizuală pentru nivelul satisfacției cu intervenția și dacă ar recomanda intervenția altor persoane.
Fezabilitatea-indicatori care țin de rata de renunțare, neprezentare la ședințe efecte adverse (Scala evenimentelor negative-Scala Efectelor Negative (NEQ, Rozenthal și Carlbring, 2015)
Nivelul anhedoniei-Temporal Experience of Pleasure Scale (TEPS), (Gard, Germans Gard, Kring, & John, 2006).
Nivelul depresiei-QIDS-SR (Rush et al., 2003) are 16 itemi care măsoară nivelul depresiei.
Apatia-subscor QIDS item 13/Indexul apatiei (3 itemi, Roberts et al., 2002).
Scala Hamilton pentru depresie (HRSD; Hamilton, 1960; Williams, 1998).
Interviul SCID 5 pentru modulele affective (SCID-5-; First et al., 2015).

Vor fi utilizate mai multe chestionare: PHQ-9 (Patient Health Questionnaire-9) pentru măsurarea nivelului depresiei, VMIQ-R pentru stabilirea abilității de imaginare(Williams et al. 2012). Scale specifice de auto-raportare pentru aprecierea imaginilor motorii asociate acțiunii și stărilor afective. BADS- The behavioral activation for depression scale (BADS, Kanter et al., 2007); Spontaneous Use of Imagery Scale (SUIS) (Reisberg, Pearson, & Kosslyn, 2003);

Mediatori: Pe parcursul intervenției se va măsura modificarea în scorurile la (1) plăcerea asociată mișcării pe parcursul intervenției (2) anhedonie (3) apatie, (4) memoria de lucru-testul digit span invers, (5) fluență verbală/acțională (6) stări afective (7) vivacitatea și ușurința imageriei motorii, (8) ruminații, (9) activarea comportamentală, (10) nivelul întăririlor.

Analiza statistică: Se va utiliza un design ANOVA mixt 2X2 pentru efectul grupului (BA, BA-PLUS și momentului evaluării pre posintervenție (intersubiecți) – asupra variabilelor rezultat: anhedonie, interes, accesibilitate, fezabilitate (nivel de semnificație: α=0.05, two-tailed). Analizele secundare pentru asigurarea robusteții rezultatelor vor include analiza diferențelor între medii (pre-post) între grupuri.

Obiective secundare: pentru moderare se va utiliza regresia liniară folosind PROCESS macro model 1 al lui A. F. Hayes (2013). Vor fi raportate media, abaterea standard, mărimea efectului și alte statistici.

VII. Descrierea activităților pe care trebuie si le desfășoare participanții (consimțământ, debriefing, etc.):

Pe parcursul cercetării, participanții completează și își exprimă acordul privind participarea (Anexa 5 din REC_UO/2019) și urmează procedura de selecție. După randomizare, participanții din grupul de tratament BA standard urmează 8 ședințe de activare comportamentală. Participanții din grupul de BA-PLUS urmează aceleași ședințe cu adăugarea intervenției T-RAC. Intervenția de activare comportamentală urmează protocolul validat în studiile de eficacitate -Martell și colaboratorii (2001; 2010) și COBRA (Richards et al., 2017).

VIII. Specificarea timpului estimat pentru completarea de către subiecți a cerințelor studiului: 60 de minute/săptămânal timp de 8 săptămâni. Sedințele de evaluare 60 minute. Variațiile de timp vor ține de nevoia terapeutică pentru participanți.

IX. Prezentarea riscurilor posibile din perspectivă morală, a situațiilor eventuale de disconfort sau stres și specificarea măsurilor luate pentru minimalizarea acestora:
Nu sunt prevăzute riscuri pentru participanți în timpul sau în urma participării la acest studiu. Participanții din grupul experimental urmează un tratament validat la scală largă atât pe populația europeană cât și nord și sud americană. Protocolul urmează ghidul BA, Martell et al., (2001;2010) și COBRA fiind verificat de către clinicieni din cadrul proiectului COBRA (McKey Dean, Voss Laura). Participanții din ambele grupuri urmează protocilul de activare comportamentală. La grupul BA plus se adaugă intervenția T-RAC. Acesată intervenție implică 10 minute de expunere Kinect (un joc Kinect timp de 10 minute) care are un nivel ridicat de acceptabilitate pe o populație diversă (non-clinică și clinică) și un exercițiu de imagerie înregistrat audio de 12 minute. Exerciții similare de imagerie au fost testate anterior atât ca intervenție de sine stătătoare (Blackwell et al., 2015) cât și ca adjuvant la BA (Patel et al., 2022) avânad o acceptabilitate ridicată. Exerciții similare de imagerie dinamică au fost testate și ele la populația normală și populație arătându-se eficiente. Din a doua ședință participanții memorează câte o activitate zilnică prin metoda augmentării motorii și își amintesc seara amintirea creată. Intervenții de reamintire au fost testate cu succes la pacienții depresivi.
În cazul apariției unor efectele negative se va anunța directorul de proiect Alexandru Tiba. Acesta va contacta participantul va decide cursul acțiunii-reorientare spre alte servicii adecvate situației și/sau consultul psihiatric. Modificările negative în starea afectivă vor fi adresate conform protocoalelor în vigoare. Participanții cu risc ridicat de suicid nu sunt eligibili pentru proiect fiind direcționați spre serviciile adecvate (cabinet psihiatric Iova Sorin/ambulatoriu psihiatrie).

Director / coordonator proiect din partea universității:
Nume: Tiba Alexandru   Data:
Semnătura:________________________
Afiliere: **Departamentul de Psihologie**
Universitatea din Oradea

CONSIMȚĂMÂNT INFORMAT

Titlul proiectului: Studiu 1 T-RAC-intervenție psihologică pentru depresie
Director proiect: Tiba Alexandru
Asistent cercetare: Sîrbu Ioana
Scopul cercetării: Studiul se desfășoară în cadrul Departamentului de Psihologie, Universitatea din Oradea și este derulat sub coordonarea dlui lector univ. dr. Alexandru Tiba.
Scopul acestui studiu vizează compararea a două intervenții psihologice pentru depresie.
Descrierea cercetării:
După selectarea în studiu, veți completa câteva chestionare si veți avea un interviu pentru a evalua starea dvs generală și abilitatea de a vă imagina. Veți fi alocat unuia dintre cele două grupe de intervenție psihologică. Un psiholog vă va contacta și veți fi programat pentru prima dintre cele 8 ședințe săptămânale. În aceste întâlniri veți urmării starea dvs și schimbările apărute, veți discuta cu psihologul activitățile antidepresive pe care le puteți face și veți găsi soluții pentru susținerea și realizarea lor. Între ședințe veți avea de efectuat activitățile antidepresive și a aplica lucrurile care vă susțin pentru a putea să le realizați. La mijlocul programului veți completa mai multe chestionare pentru a urmări schimbările realizate. La fel, la o săptămână după terminarea programului veți urma evaluarea rezultatelor precum și la 3 luni după program. Ședințele vor fi inregistrate audio și verificate aleator pentru asigurarea fidelității intervenției.Participarea la program înseamnă acordul dvs de a înregistra ședințele.
Vă asigurăm confidențialitatea participării și a informațiilor oferite pe parcursul studiului, precum și siguranța procedurilor utilizate. Datele dumneavoastră vor fi utilizate doar în scop de cercetare.
Întrebările nu au caracter defăimător şi nici conținuturi care pot produce emoții negative.
Evaluarea nu are un caracter de evaluare personală și nu se vor emite judecăți de valoare pe baza acestor răspunsuri. În orice moment puteți renunța la participarea în cercetare. Puteți opta pentru informarea dvs cu privire la rezultatele evaluării bifând căsuța corespunzătoare solicitării furnizării informații cu da. În urma solicitării vi se va trimite situația dvs și recomandările adecvate pentru evaluarea suplimentară. După ce sunteți de acord și vă dați consimțământul pentru participare, veți putea participa la cercetare.
Riscurile potențiale: Nu sunt riscuri în urma participării la acest studiu.
Beneficii potențiale: În cazul în care optați pentru informarea cu privire la starea dvs. veți primi un email în care vor fi trecute rezultate cu privire la aceasta.
Costuri / Compensații: De asemenea nu sunt beneficii financiare sau materiale în urma participării la studiu. Participarea la acest studiu nu este compensată financiar sau material
Persoana de contact: alexandrutiba@gmail.com Dacă aveți întrebări suplimentare despre cercetare, oricând puteți lua legătura cu directorul de proiect la email alexandrutiba@gmail.com.
Confidențialitate: Informațiile colectate vor fi păstrate sub confidențialitate, în conformitate cu legislația în vigoare. Datele vor fi analizate în condiții de anonimat și vor fi accesate doar de către echipa de cercetători. În baza Regulamentului (UE) 2016/679 privind protecția persoanelor fizice în ceea ce privește prelucrarea datelor cu caracter personal și privind libera circulație a acestor date și de abrogare a Directivei 95/46/CE (Regulamentul general privind protecția datelor) și ale Legii nr. 506/2004 privind prelucrarea datelor cu caracter personal și protecția vieții private, datele furnizate de dvs. în prezentul studiu vor fi folosite doar pentru scopul specificat de studiere a rolului cognițiilor și emoțiilor și a interacțiunilor dintre acestea în riscul pentru tulburări emoționale și emoții disfuncționale și analizare a acestor factori. Analizele statistice vor fi efectuate la nivelul eșantionului și nu la nivel individual.
Participare voluntară: Participarea la această cercetare este voluntară. Decizia de a nu participa sau de a renunța pe parcurs nu va duce la nicio penalizare, amenzi sau pierderea beneficiilor dvs. Totuși dacă doriți retragerea din studiu veți fi întrebat legat de motivele care au stat la baza retragerii dvs. Participarea poate fi întreruptă dacă: (1) starea dvs se înrăutățește, (2)apar situații legate de neeligibilitatea pentru studiu (3) nu realizați sarcinile presupuse pentru desfășurarea programului sau nu participați la 3 întâlniri.
Aprobarea Comisiei de Etică
Această cercetare a fost analizată și aprobată de Comisia de Etică a Universității din Oradea.

Semnături

Data__________________	Data_______________
Director de proiect	*Participant la studiu*
Nume__________________	Nume__________________
Semnătura______________	Semnătura______________
